# Shaken Baby Syndrome/Abusive Head Injury: The Role of Expert Witness Testimony and a Recent Case Development

**DOI:** 10.1007/s11673-025-10422-x

**Published:** 2025-05-20

**Authors:** James Tibballs, Neera Bhatia

**Affiliations:** 1 Department of Paediatrics, The University of Melbourne, Parkville Campus, Melbourne, VIC Australia; 2 School of Law, Faculty of Business and Director of Law, Health and Society Research Unit, Deakin University, Geelong, Australia

**Keywords:** Law, Children, Shaken baby syndrome, Expert witness testimony, Witness opinion, Health law, Criminal law

## Abstract

The triad of clinical signs, (extensive bilateral retinal haemorrhages, subdural haematoma, and encephalopathy) is regarded by some expert witnesses as pathognomonic proof that an infant was deliberately shaken and head injured (shaken baby syndrome / abusive head injury). However, that view is controversial since scientific evidence does not support the diagnostic accuracy of the triad. In contrast to previous cases, a Victorian Supreme Court jury found an accused not guilty of homicide of a one-month-old infant afflicted with the triad. Prosecution witnesses were heavily criticized for failing to provide impartial testimony and to abide by Supreme Court expert evidence rules. We argue that there is a need to reassess the manner in which expert witness testimony is considered by the courts in shaken baby cases where injury has caused the death of the infant.

## Introduction

For decades in Australia, courts have adopted an orthodoxy—with few exceptions (*R v Klamo* [2008] VSCA 75; *R v Lee* [2001] ACTSC 133)—of convicting defendants in shaken baby syndrome/abusive head trauma (SBS/AHT) cases based on opinions of forensic medicine experts (Brook and Nott 2023), but in a recent Victorian Supreme Court case, a jury found a man not guilty of deliberately shaking an infant causing death.^1^ The decision in this case is a watershed moment in the Victorian legal landscape, given that the court had dismissed a recent shaken baby syndrome (SBS) appeal case and had convicted the accused in a prior case (*Vinaccia v The Queen* [2022] VSCA 107 (‘*Vinaccia*’); *R v Rowe* [2018] VSC 490). In all cases, the infants presented with a “triad” of clinical signs comprising subdural haematoma, bilateral severe retinal haemorrhages, and encephalopathy, without other injuries. In such cases, the opinions of medical experts, unsupported by scientific evidence, should have been ruled inadmissible (Edmond 2020).

In this article, we highlight the possible reasons for disparate outcomes of the Victorian Supreme Court trials of SBS. Shaken baby syndrome is also known as abusive head trauma (AHT), non-accidental head injury (NAHI), and more recently as retinodural haemorrhage of infancy (Guthkelch 2012; Squier 2023). We emphasize the role and importance of impartiality of medical expert witness opinion, especially in cases of SBS/AHT where controversy exists. We consider the evolutionary role of expert witness opinion testimony in litigated cases of SBS while focusing on its requirements under Victorian legislation and Supreme Court rules.

Two anomalies in medicine and law are evident when diagnosing SBS/AHT for judicial decisions. The first is that the scientific evidence that supports the triad as being due to SBS/AHT is of low to very low and of poor quality, yet such evidence is relied upon to diagnose deliberate injury. A 2003 Australian systematic review (Donohue 2003) found there was inadequate scientific evidence to form a conclusion on any matters pertaining to SBS, including diagnosis. A more recent (2016) systematic review by the Swedish Council on Health Technology Assessment (SBU) of the medical literature pertaining to the triad of clinical signs in diagnosing SBS/AHT, concluded that there was insufficient scientific evidence to support the triad’s diagnostic accuracy. The report attracted severe criticism from the Royal College of Paediatrics and Child Health, which stated it had methodological flaws and recommended that it be withdrawn (Debelle et al. 2018). Their opinion was that a search of the scientific literature would identify high-quality scientific studies which have improved the identification and understanding of AHT, but no articles were cited. The Swedish investigators had examined 3,773 journal abstracts, reducing this list first to 1,065 full texts and then to only two articles (Vinchon et al. 2010; Adamsbaum et al. 2010) after excluding the others as evidencing circular reasoning (Lynøe and Eriksson 2018) and methodological problems. These two articles, rated as being of moderate quality by the investigators, supported the concept that shaking could cause the triad of injuries; however, they were undertaken in part on subjects who had confessed while in police custody.

We (Tibballs and Bhatia 2024) subsequently assessed these two studies as being of low-quality due to multiple biases and contamination by reliance on unreliable confessions (Rossant and Brook 2022; Findley 2023a, b). In other words, all the scientific evidence pertaining to SBS/AHT is of low to very low quality and is thus unreliable (Tibballs and Bhatia 2024). Medical opinions arising from unreliable evidence cannot be anything but themselves unreliable. Evidence thus categorized is not given credence in other fields of medicine. It does not even rate in the hierarchy of scientific evidence—why so in the field of paediatric forensic science? Expert forensic medical opinion based on such evidence should be ruled inadmissible by the court (Edmond 2020).

The second anomaly is that in trials of SBS/AHT comprising only the triad on medical opinion, deliberate child abuse is assumed by default, not proven by the prosecution. Although the strategy of making a diagnosis by exclusion of others *may* suffice in medicine, it does not apply to a judgement in law which requires that the burden begins and always remains on the prosecution to prove the defendant’s guilt beyond a reasonable doubt. Stephen Cordner, the former director of the Victorian Institute of Forensic Medicine, remarked— referring to guidelines for admitting forensic evidence to court—that “… a category we have to be careful of is the diagnosis—or coming to conclusions of any sort—by exclusion” (Cordner 2012). An analogy would be one of two criminal suspects having an ironclad alibi, and therefore the second person being assumed guilty by default. A similar logic has operated to convict suspects of SBS/AHT merely because no other explanation has been recognizable.

## The Victorian Evidence Act: Who can Be an Expert Witness?

In Victoria, expert witness opinion is governed by the *Evidence Act 2008*. Under Sect. 76, opinions are generally considered inadmissible. However, exceptions apply in the circumstances outlined in Sect. 79, “where a person has specialized knowledge based on the person’s training, study or experience.” Here, the general rule of the inadmissibility of opinion does not apply to “evidence of an opinion of that person that is wholly or substantially based on that knowledge.”

Notably, under Sect. 79(1) of the Act, any expert opinion proffered must satisfy three criteria: (1) the expert must possess specialized knowledge; (2) that knowledge must be based on relevant training, study, or experience; and (3) the opinion must be based on that specialized knowledge—a nexus criterion. The opinion that is offered must be wholly or substantially based on the expert’s claimed specialized knowledge. Each opinion must be examined for its basis in the expert’s relevant training, study, or experience and may be disqualified or ruled inadmissible if the nexus cannot be established. Additionally, “specialized knowledge” need not be derived from tested or reliable fields of study using accepted and proven methods. When an expert opinion is contentious or based on new or cutting-edge science, reliability as a criterion is paramount and is ultimately a question for the court to decide.

A person who holds themselves out to be an expert is obliged to disclose the facts or assumptions on which an opinion is based. The facts and assumptions “must be capable of proof by admissible evidence; and evidence must be admitted to prove the facts and assumptions upon which the opinion is based.” The opinion must be based on known evidence and reliable. In Australia, there has been some debate about the appropriate test for reliability of expert knowledge in new and developing fields. The courts have sought to acknowledge and recognize circumstances where a body of expert knowledge has “general acceptance” in the “relevant, usually scientific discipline” (*Honeysett v The Queen* (2014) 253 CLR 122). However, while Australian courts are required by legislation to consider whether opinions are based on “specialized knowledge” and if such opinion is based on “training, study or experience,” they are not required to consider the reliability and validity of an opinion (Edmond 2020). Although a medical expert is required (see below) to furnish a reliable and valid opinion, the court is not required to ascertain its reliability and validity (Edmond 2020), and the legislation does not require adherence to court codes of practice by expert witnesses. Instead, the “specialized knowledge” based on “training, study or experience” of an expert is a “short cut” for what should be a court-based assessment of the reliability and validity of evidence (Edmond 2020) which Cordner and Woodford (2020) point out is made in Australia by the jury.

## The Supreme Court of Victoria Practice Note for Expert Evidence in Criminal Trials

As described in a practice note^2^ originating after *R v Klamo* (Cordner 2012; Brook and Nott 2023), experts have duties to the court to enhance the *quality and reliability* of expert evidence relied upon by the prosecution and the accused in criminal trials in the Supreme and county courts under the *Crimes Act 1997*.

Section 4 describes an expert’s duty to the court:

- 4.1 An expert has an overriding duty to assist the Court impartially, by giving objective, unbiased opinion on matters within the expert’s specialised knowledge.
- 4.2 This duty overrides any obligation to the commissioning party or to the person by whom the expert is paid.
- …
- 4.4 An expert is not an advocate for a party even when giving testimony that is necessarily evaluative rather than inferential.
- Section 6 describes what must be included in expert reports:
- 6.1 …
- (b) an acknowledgement that the expert has read the Practice Note and agrees to be bound by it;
- (c) whether and to what extent the opinion(s) in the report are based on the expert’s specialised knowledge, and the training, study experience on which that specialised knowledge is based;
- (d) the material, observed facts, reported facts, assumed facts and other assumptions on which each opinion expressed in the report is based (a letter of instruction may be annexed);
- (e) (i) the reasons for,
  - (ii) any literature, research or other materials or processes relied on in support of,
  - (iii) a summary of—
- each such opinion
- (f) (if applicable) that a particular question, issue or matter falls outside the expert’s specialised knowledge;
- (g) any examinations, tests or other investigations on which the expert has relied, identifying the responsible laboratory by which, and the relevant accreditation under which, the examination, test or other investigation was performed;
- (h) a declaration that the expert has made all the enquiries and considered all the issues which the expert believes are desirable and appropriate, and that no matters of significance which the expert regards as relevant have, to the knowledge of the expert, been withheld;
- (i) any qualification of an opinion expressed in the report, without which the report would or might be incomplete or misleading
- (j) any limitation or uncertainty affecting the reliability of
  - (i) the methods or techniques used; or
  - (ii) the data relied on—
- to arrive at the opinion(s) in the report; and
- (k) any limitation or uncertainty affecting the reliability of the opinion(s) in the report as a result of—
  - (i) insufficient research; or
  - (ii) insufficient data.
- 6.2 Where an expert is aware of any significant and recognised disagreement or controversy within the relevant field of specialised knowledge, which is directly relevant to the expert’s ability, technique or opinion, the expert must disclose the existence of that disagreement or controversy.

This practice note along with the sections discussed above in the *Evidence Act 2008* are of importance and relevance to the discussion below in our commentary of the SBS/AHT cases.

## Cases of SBS Before the Victorian Supreme Court

We analyse three recent cases of SBS/AHT with divergent outcomes in the Supreme Court of Victoria and the Court of Appeal and specifically draw attention to how medical expert witnesses failed to follow or succeeded in following the practice note outlined above, with attendant consequences. It is noted that in 2021 a Victorian coroner, after hearing medical opinions in favour of a non-accidental cause and evidence in favour of an accidental cause, was unable to determine whether the triad of injuries leading to the death of a seven-month-old infant was caused by a fall from a high-chair or whether it was due to abusive head trauma.^3^ The hearing was preceded by a request to the coroner from a forensic expert to disallow consideration of scientific evidence suggesting an accidental cause.

### R v Rowe

In 2015, Joby Rowe drove the mother of baby Alanah to work. On her return, the mother noticed that Alanah, three months-of-age, was pale and gasping for breath. She performed cardiopulmonary resuscitation (CPR) while waiting for emergency services. Paramedics were able to re-establish cardiac output. Joby informed the paramedics that he had attempted to give the baby a bottle feed when she began coughing, gasping, and then vomiting. She had a nosebleed and fell backwards and became unconscious. He denied shaking the baby and there were no external injuries. Two weeks later, the baby’s condition deteriorated, and life support was withdrawn. At the trial, two medical practitioners stated that in their opinion the death was the result of injuries caused by violent shaking with or without impact on a soft surface. Additionally, neither doctor had considered any other possible reasonable explanation. A third doctor stated that “the widespread extent of the retinal haemorrhages was suggestive of significant head trauma.” No defence was offered, and no scientific medical evidence was adduced by prosecution witnesses to support their opinions, in contravention of their duty to the court. In 2018, Joby Rowe was convicted of homicide and sentenced to nine years imprisonment. This conviction was criticized in a published article that pointed out that it lacked a scientific basis, but the article was withdrawn upon pressure exerted by prosecution witnesses, providing an example of how dissent is silenced (Brook 2023a, b).

### Vinaccia v The Queen

In 2019, Jesse Vinaccia was convicted of homicide caused by his handling and shaking to death of sixteen-week-old baby Kaleb in a manner that was either unlawful and dangerous or criminally negligent. At the trial, it was stated that the infant had clinical signs of the “triad,” which in the absence of any alternative explanation was attributed by expert witnesses to deliberate forceful shaking. Vinaccia was sentenced to eight and a half years’ imprisonment.

In 2023, he appealed the conviction in the Victorian Supreme Court of Appeal. The appeal was heard before a panel of three judges and focused on five grounds for appeal. The main grounds for appeal were that the clinical diagnosis of inflicted head injury based on the “triad” of signs was derived from improper science (also referred to as “junk science”) and that new or fresh evidence was available. That new evidence included the Swedish review (SBU 2017) published before the trial—which had concluded that there was insufficient scientific evidence on which to assess the diagnostic accuracy of the triad in identifying traumatic shaking. We assessed this study as low quality (Tibballs and Bhatia 2024), but it nonetheless highlighted a controversy which, although known to and taught academically by the prosecution medical experts, had not been brought to the notice of the trial court, in defiance of their duty. In addition, since the prosecution witnesses did not adduce any scientific evidence, the reliability of their opinions was not established as required under the Supreme Court’s practice note. The prosecution witnesses claimed, in support of a non-accidental injury, that there was a “body of evidence … such as retrospective studies … that has not been on a base of circular logic” (Vinaccia, 414). However, the witnesses did not adduce such evidence. Absent any eye-witness status, it is thus difficult to understand how prosecution witnesses were able to form a view that deliberate abuse had occurred. The authors of the Swedish systematic review appeared for the defence at the appeal but their evidence was assessed by the majority judges as “well short of establishing that the applicant should not have been convicted and there had been a miscarriage of justice” (Vinaccia, 412). It is surprising that available Australian defence witnesses were not called. The appeal was refused. This case is an illustration of the determinative power of medical opinion in the face of insufficient evidence, the lack of adherence by forensic experts to court rules and the lack of the court’s application of the rules (Edmond 2020).

### DPP v DCT

In 2017 a one-month-old infant become floppy and unresponsive while in the brief care of the accused. CPR was applied by both parents. Ambulance officers found the infant pulseless but managed to restore circulation with injections of adrenaline and mechanical ventilation via endotracheal intubation. However, a prolonged hypoxic-ischaemic brain injury had occurred. In the hospital, the triad of injuries was identified. Unfortunately, cerebral oedema worsened and resulted in brain death as diagnosed by radionuclide perfusion scan. At autopsy, no external injuries or fractures were noted but additional relevant findings included central parietal-occipital and right-sided parietal subgaleal bruising (caused by trauma), absent intact bridging veins associated with bilateral subdural haematomas, cervical spinal cord ischaemia, subarachnoid haemorrhage and haemorrhage around the cervical nerve roots and dorsal root ganglia, which like the triad of signs, were all considered possible from a short fall by defence witnesses.

While the rationale for the jury’s verdict of not guilty is unknowable, prosecution expert witnesses were criticized heavily during the trial for predetermined bias and beliefs of guilt of the accused, for failing to present an impartial view of the cause of the infant’s triad of signs, for failing to consider alternative explanations for the infant’s injuries, for failing to declare the controversial nature of the diagnostic accuracy of the triad signs, and for colluding with the police. In addition, the radiologist selected by the forensic service was criticized as being biased in favour of diagnosing head trauma as non-accidental rather than accidental, as was the prosecution for endeavouring to exclude testimony from a defence witness. In contrast, defence witnesses presented scientific evidence that in their opinion deliberate and accidental causes of the injuries could not be differentiated. The jury returned a verdict of not guilty.^4^

## Discussion

The consideration of the triad of clinical signs as the only and pathognomic cause of SBS/AHT is the source of ongoing controversy in medico-legal literature (Findlay et al. 2012; Cordner et al. 2012; Roygardner et al. 2020; Findley et al. 2023). From the 1970 s, the identification of the triad of injuries in infants (Caffey 1972; Guthkeltch 1971) has been regarded as the default dogmatic proof of recent deliberate non-accidental trauma despite the founder of the diagnosis stating it is unproven (Guthkelch 2012). However, since shaking alone cannot produce the triad of injuries (Duhaime et al. 1987) the requirement of additional direct head trauma was added by the American Academy of Pediatrics in 2009 necessitating a change in the appellation of the syndrome to “abusive head trauma.” (Christian et al. 2009). In addition, in 2015 the American Academy of Ophthalmology declared that when no other explanation can be identified, if multiple retinal haemorrhages are accompanied by retinal perimacular folds and schisis cavities, the diagnosis of non-accidental trauma can be diagnosed with confidence (American Academy of Ophthalmology 2015).

In 2018, a “consensus statement” (Choudhary et al. 2018) —not a scientific study but a policy statement—claimed that there was no controversy and no reliable medical evidence that the constellation of injuries can be mimicked by other conditions but also advised that the workup must exclude medical diseases that can mimic AHT and claimed that severe intracranial injury from short falls is rare. This consensus statement was roundly criticized by prominent legal and medical academics as being a “feigned consensus” (Findley et al. 2019). They showed, with many examples, that a live controversy does indeed exist about the diagnostic value of the triad for SBS/AHT and maintained that under traditional principles of evidence law physicians should not be permitted to “diagnose” abuse in court as opposed to identifying specific medical symptoms and signs. Expert witnesses should not be permitted to “overstep” their specialized knowledge.

Despite such authoritative statements, the aetiology of the triad of injuries is controversial, being dependent on non-conclusive expert medical opinion and inevitably by opposing opinion thus making fair judgement difficult. The diagnostic value of the triad for non-accidental injury has been questioned with the recognition that low-impact injuries, such as a fall from the sitting or standing position, from household furniture, or from a carer’s arms, may also cause the triad of injuries (Aoki and Masuzawa 1984; Chadwick et al. 1991; Brook et al. 2024). Other common conditions causing the features of SBS include subdural hematoma from birth trauma, benign external hydrocephalus, and thrombosis of a cortical vein or sagittal sinus (Miller 2023), while conditions which predispose to the findings of SBS with minimal trauma include osteogenesis imperfecta and Ehlers-Danlos syndrome (Echenne 2023). The mechanism creating subdural haematoma is purported to be an acceleration-deceleration movement of the brain within the skull which shears off the bridging veins as they traverse the subdural and subarachnoid spaces from the brain surface into the dura mater. Such a mechanism could operate when the infant’s head traumatically strikes (or is struck by) a solid object. The intracranial shearing force created by the head striking a solid object (e.g., the floor during a fall) is many times more than the forces created by shaking (Ommaya et al. 2002). The pathogenesis of retinal haemorrhages is the numerous causes of intracranial hypertension (Miller 2023).

Although the triad causing death is a medical diagnosis and is claimed not to be a legal determination by adherents to SBS/AHT doctrine (Choudhary et al. 2018), it does in reality portend a charge of homicide—on the basis of medical expert opinion alone. This position has created medical and legal controversy.

According to Lynøe and Eriksson (2019), the triad has become synonymous with malevolent intent because of our universal desires to prevent child abuse and to seek medical assistance for an injured child. These laudable ethical intentions have allowed cognitive and advocacy biases to overcome scientific rigour such that low-level scientific medical evidence has been falsely construed to support the inculpation of caregivers. The result is that cases of AHT are over-estimated (Lynøe and Eriksson 2019) or are over-diagnosed (Brook 2023a, b).

Some countries have already abandoned the concept that the identification of the triad in the absence of other explanations automatically implies SBS/AHT. In the United States, after a review of numerous cases, it was concluded a decade ago that the diagnostic triad does not prove beyond a reasonable doubt that an infant was abused or that the last person caring for the infant was responsible (Tuerkheimer 2014). Numerous convictions have been overturned based on emerging science (new evidence) or trial errors (Findley 2023a, b). In Canada the triad is not accepted as a definitive diagnosis of abuse, but it remains a “strong pointer” to it (Ebbs 2011). In 2021, the U.K. Director of Public Prosecutions (re)issued advice for prosecutors dealing with complex conflicting expert evidence in putative non-accidental head injury (NAHI) cases with the triad of injuries only. Considering *R v Henderson*, *R v Butler*, *R v Odeyiran* [2010] Cr App R 24, this advice stated that the jury should be reminded that.

… there are limits to the extent of knowledge and no conclusion should be reached without acknowledging the possibility of an unknown cause emerging into the light of medical perception and that the mere exclusion of every possible known cause does not prove the deliberate infliction of violence. (21)

Moreover: “… where the only evidence available is the triad of pathological features … Such evidence is rarely conclusive of NAHI and prosecutors should look for other supporting evidence” (21). However, in France as of 2017 a diagnosis of NAHI caused by shaking is considered certain in the absence of another explanation in cases of multifocal subdural haematoma or multifocal subdural haematoma with any retinal haemorrhage or unifocal subdural haematoma with cervical and/or spinal cord lesions. It is also considered likely in cases of multifocal subdural haematoma without any other lesion or unifocal subdural haematoma with intraretinal retinal haemorrhage limited to the posterior pole or with retinal haemorrhage affecting the periphery and/or several layers of the retina, whether unilateral or bilateral (Haute Autorité de Santé 2017). However, the guideline is currently being revised (Haute Autorité de Santé 2017; Rossant and Etrillard 2023; Rossant et al 2024).

The basic difficulty in differentiating deliberate from accidental injury is that the medical evidence consists necessarily only of cases series of victims, which in the hierarchy of scientific evidence is of low level and is usually of poor quality due to multiple biases, such that reliable conclusions cannot be made (Tibballs and Bhatia 2024). The upshot is that judicial enquiries in cases of SBS rely heavily upon the opinions of medical experts which can only be supported at best by such unreliable medical evidence. However, in this context, if medical opinions are restricted to physicians who believe the accused is guilty, and if no or inadequate defence is offered (*Vinaccia*; *R v Rowe* [2018] VSC 490), the court will inevitably find the accused guilty, as in the initial cases in this small Victorian series suggests.

Such evidence and expert opinion derived from it should be ruled inadmissible by the court (Edmond 2020). For medical expert opinion to be admissible, it must be the product of methodology that is reliable and is reliably applied (Pakes 2023). The methodology must include the establishment of general causation which in the condition of SBS/AHT means that shaking must have been shown to cause the triad of clinical signs, but this has never been shown. Clinical decision-making is not a valid methodology for establishing general causation. Put simply, the exclusion of other possible conditions to explain a triad of signs is not valid legal methodology to diagnose abusive injury, is thus legally unreliable and inadmissible (Pakes 2023).

*DPP v DCT* 2024 can be heralded as a watershed moment in Victoria in these types of cases involving the triad of injuries unaccompanied by other injuries. It is the first Victorian case in which the accused was found not guilty of causing the death of an infant who had died solely from the triad of injuries.^5^

While the circumstances of this case are tragic, it was concluded that the cause of death was accidental, not criminal. In contrast to the previous cases, it appears that expert defence witnesses adhered with their duties to the court.

This case shines the spotlight on the importance and the need for independent and impartial expert witness testimony. Cases of SBS have typically relied heavily on the testimony of medical experts; however, that testimony must not be biased or based on predisposed beliefs or confirmation bias. The courts and judges in these cases must be able to gauge the most relevant and appropriate findings, and rule whether evidence based on opinion is admissible. Expert witnesses must comply with *Practice Note SC CR 3*; not doing so will mean they are in breach and will bring the legitimacy of the entire case into question. Taking a biased or partisan view has significant implications for the lives of the accused and their families.

## Conclusion

In the most recent Victorian recent case, *DPP v DCT*, the dogma of guilt associated with the triad of injuries alone was refuted after expert defence witnesses adhered to the court rules. This is a change compared to previous cases in which only expert opinion, unsupported by science, determined outcomes. Expert witnesses are reminded that their duty is to the court and not to either party in a dispute. They are advised to present unbiased and impartial opinions so that the court may fairly determine the outcome. Moreover, expert witnesses should present evidence to support their opinion and declare matters of medical controversy or uncertainty relevant to their areas of specialized knowledge. The topic of SBS/AHT is currently receiving considerable public attention, it is timely for the legal and medical fraternity to reconsider the controversies that surround this issue. Given the recent decision in *DPP v DCT* there is a possibility of previous cases being reviewed and over time the court decision might lead to a precedent for future cases.

## Footnotes

1 Doogue George Defence Lawyers. Transcripts of court proceedings DPP v DCT 2024 (‘Transcript DPP v DCT’). This case is subject to an identification suppression order until July 31, 2025, and not available to the public.

2 Supreme Court of Victoria. *Practice Note SC CR 3. Expert Evidence in Criminal Trials*, September 2017.

3 Coroners Court of Victoria. *Inquest into the death of Mathew Jameel*. COR 2018 2777.

4 Transcript DPP v DCT. Also note the case of *Jesse Harvey v The King* [2023] VSCA 219. The jury found the applicant guilty of one count of recklessly causing serious injury to his seven-week-old son. It was determined that the infant suffered a subdural haematoma, but not the “triad of injuries.” The applicant was refused an appeal. However, the judgement highlights some of the concerns we have raised where there is heavy reliance on expert witness testimony.

5 In *R v Klamo*, an infant died from a subdural haematoma. At trial the accused was judged guilty by causing death by shaking but this verdict was overturned on appeal on the basis that it could not be shown that it was shaking rather than re-bleeding into a pre-existing lesion acquired at birth which caused death).
